# Mutations of *MACF1*, Encoding Microtubule-Actin Crosslinking-Factor 1, Cause Spectraplakinopathy

**DOI:** 10.3389/fneur.2019.01335

**Published:** 2020-01-15

**Authors:** Lulu Kang, Yi Liu, Ying Jin, Mengqiu Li, Jinqing Song, Yi Zhang, Yao Zhang, Yanling Yang

**Affiliations:** ^1^Department of Pediatrics, Peking University First Hospital, Beijing, China; ^2^Euler Genomics, Beijing, China

**Keywords:** myopathy, MACF1, whole exome sequencing, spectraplakinopathy, neuromuscular disorder

## Abstract

As a member of spectraplakin family of cytoskeletal crosslinking proteins, microtubule-actin crosslinking factor 1 (MACF1) controls cytoskeleton network dynamics. Knockout of *Macf1* in mice resulted in the developmental retardation and embryonic lethality. Spectraplakinopathy type I, a novel neuromuscular condition characterized by periodic hypotonia, lax muscles, joint contracture, and diminished motor skill, was reported to be associated with heterozygous genomic duplication involving the *MACF1* loci, with incomplete penetrance and highly variable clinical presentation in a single pedigree. In this study, parental-derived compound heterozygous novel missense mutations of *MACF1*, c.1517C>T (p.Thr506Ile) and c.11654T>C (p.Ile3885Thr), were found to co-segregate with disease status in two affected brothers presenting with progressive spastic tetraplegia, dystonia, joint contracture, feeding difficulty and developmental delay. We speculated that *MACF1* mutations cause spectraplakinopathy inherited in an autosomal recessive manner. Our clinical findings expanded the phenotype of this neuromuscular disorder and provided new insights into the function of MACF1.

## Introduction

Spectraplakins act as cytoskeletal crosslinkers by coordinating with three types of cytoskeletal filaments, including microfilaments (F-actin), microtubules, and intermediate filaments, in multicellular organisms (1). The interconnected network of cytoskeletal filaments determines cell shape, structure, differentiation, polarization, division, adhesion, migration, intracellular trafficking, and organelle locomotion (1). Mammalian spectraplakins include two known members: microtubule-actin crosslinking factor 1 (MACF1; also known as actin crosslinking factor 7, ACF7) (2) and bullous pemphigoid antigen 1 (BPAG1), which are evolutionarily conserved in multicellular organisms (3).

Human MACF1 (also termed as macrophin 1 or trabeculin-alpha) was originally reported as an actin-crosslinking protein (2) and is encoded by *MACF1*, which is localized on chromosome 1p34 and comprises at least 102 exons and spans over 270 kb (4). The mRNA of *MACF1* can be detected in the brain, heart, lung, liver, stomach, kidney, skin, neural, skeletal muscle, and bone tissues (5). At over 600 kDa, MACF1 is a gigantic protein, which shares characteristics of both the spectrin and plakin families. Structurally, MACF1 comprises an N-terminal domain, rod domain, and C-terminal domain. Six different MACF1 isoforms produced via alternative splicing and differential promoter usage have been identified, including MACF1a1, MACF1a2, MACF1a3, MACF1-4, MACF1b, and MACF1c.

Evidence suggests that MACF1 plays vital roles in organisms. *Macf1* knockout mice or conditional knockout in specific tissues were used to explore the potential role of *Macf1*. The results showed that *Macf1* was essential for embryo development (5) and was indispensable for maintaining the neuronal system, bone, colon, cardiomyocyte function, and skin integrity (5). For genetic diseases in humans, in addition to the recently reported neuromuscular disorder (Spectraplakinopathy type I), it was discovered that *MACF1* is a candidate gene for human schizophrenia and was identified as a risk gene for Parkinson's disease. In addition, aberrant expression of MACF1 is also related to human cancers, such as breast cancer, colon cancer, liver cancer (6, 7), lung cancer, glioblastoma, and renal cell carcinoma (8, 9).

The loss-of-function (LoF) allele of *MACF1* is under strong purifying selection (ExAC LoF pLi = 1.00, http://exac.broadinstitute.org/), suggesting that null alleles of *MACF1* are highly pathogenic. Since its first description in 1995, only one pedigree with genomic duplication of *MACF1* has been described (10). The *MACF1* genomic duplication interfered with gene expression and was associated with a clinically variable neuromuscular disorder. In the present study, we described a Chinese quartet pedigree with two offspring exhibiting phenotypically similar neuromuscular disorders that co-segregated with parental-derived compound heterozygous mutations in *MACF1*. We speculated that *MACF1* mutations cause spectraplakinopathy inherited in an autosomal recessive manner.

## Case Report

The proband is the second son of healthy parents. He was born at full term with a birth weight of 3,300 g after an uneventful pregnancy. He was referred to the Department of Pediatrics, Peking University First Hospital, at the age of 2 years and 1 month because of psychomotor retardation. His physical growth was normal. His body height was 85 cm (−2.0 SD), his weight was 13 kg (0 SD), and his head circumference was 48 cm (0 SD). He was able to raise his head at the age of 5 months old, turn over at 1 year old, and sit up at 2 years old. He could not walk at the age of 2 years and 1 month. His parents complained that he had poor eye fixation and seldom fixated on one object. Physical examination revealed joint contracture of knees, hypotonia of lower limbs, and normal reflexes. The manual muscle testing of the distal muscle was M2 (active movement, with gravity eliminated), proximal muscle M2 (active movement, with gravity eliminated), and axial muscle M3 (active movement against gravity). The degree of disability measured by Modified Rankin Scale was grade 4 (Moderately severe disability).

High resolution chromosomal karyotyping of the proband showed a normal male karyotype (46, XY). His blood-free carnitine was mildly decreased (12 μM, reference range 20–60 μM). The blood acylcarnitine profile and urinary organic acids were normal. Other tests including routine blood test, blood gas analysis, plasma ammonia, myocardial zymogram (lactate dehydrogenase, creatine kinase, creatine kinase muscle b), and liver function (aspartate transaminase, alanine transaminase) revealed no remarkable abnormalities. Peripheral leukocyte lysosomal enzyme activities, such as α-galactosidase A, α-glucosidase, β-galactosidase, β-glucocerebrosidase, and α-L-iduronidase, were normal. Further examination revealed elevated urinary glycocyamine and creatinine (374.27 mmol/mol vs. normal range 10–280 mmol/mol cr) and the creatine creatinine ratio (1,517.43 mmol/mol vs. normal range 10–1,500 mmol/mol cr), respectively. Brain magnetic resonance imaging (MRI) showed delayed myelination of white matter.

The proband's elder brother was 8 years old and had more severe symptoms. His psychomotor development was much delayed. He did not reach any developmental milestones. He still could not raise his head, sit up, and stand when he visited our hospital at the age of 8 years. He almost manifested a persistent vegetative state because he barely reacted to the outside world. He had trouble talking, chewing, and eating, and he showed joint contracture of the bilateral knees. His manual muscle testing score of overall muscle groups was M0 (no contraction) or M1 (flicker or trace of contraction). His degree of disability evaluated by the Modified Rankin Scale was grade 5 (Severe disability). Additionally, he was presented with intractable epilepsy from the age of 1 year. Electroencephalogram showed theta waves, delta waves, and sometimes sharp waves and sharp slow waves. His blood amino acids and acylcarnitine profiles were normal. Chromosome microarray analysis showed no abnormalities. His brain MRI revealed white matter dysplasia.

## Materials and Methods

### Quartet Whole Exome Sequencing

Whole exome sequencing (WES) was performed on the two brothers and their parents. Briefly, genomic DNA was extracted using a standard protocol (11), quality-controlled, and captured using the Integrated DNA Technologies consensus coding sequence (39 Mb) exome panel. The post-capture library was sequenced on an XTEN sequencer according to the manufacturer's instructions (Illumina, San Diego, CA, USA). Reads were aligned to the reference human genome (GRCh37) using Burrows-Wheeler Aligner (v.0.5.9-r16). The average sequencing depth was >100-fold, exons at over 30× coverage comprised 99.83–99.85% of all designed probe regions, and gaps (with coverage as low as 0×) made up 1.6–1.9% of all target intervals. The aligned data were then processed using Freebayes (12) to call variants. A filter of SAR (Number of alternate observations on the reverse strand) > 1, SAF (Number of alternate observations on the forward strand) > 1, QUAL (quality) > 20, DP (depth) > 30 was applied to the variants for initial filtration. A second filtration was applied with a stringent population frequency of AC ExAC EAS (East Asian allele count in ExAC) <2, Hom ExAC = 0, 1,000 Genome AF < 0.001, gnomAD AF <0.001, and an in-house database with a phenotype-related frequency. Mutations passing the population frequency filter were then subjected to annotation with Spidex (http://tools.genes.toronto.edu/), SnpEff (http://snpeff.sourceforge.net/SnpEff_manual.html), and SCADA (a supervisory control and data acquisition system). Structural variations were called using Delly (a tool to analysis structure variation) and copy-number variations (CNVs) were called using EulerCNV (an in-house algorithm using similar-strategy captured data as reference and performs circular segmentation based on the Z-value and BAF). A summarized variation table consisting of single-nucleotide variants (SNVs), CNVs, and structural variants (SVs) were then passed to the inheritance mode check. The Mendelian inheritance vector was calculated using a logical operator from SnpSift. The final variation table was aggregated to a gene-mutation-burden table. A natural language processing-based search program Langya was applied to match each gene name in the gene-mutation-burden table with a clinical phenotype, resulting in a likelihood score for prioritizing the mutation list.

### Sanger Sequencing

Sanger sequencing and polymerase chain reaction (PCR) (13) were used to confirm the compound heterozygous variations identified by WES in the *MACF1* gene. Primers used for amplification of coding exons and flanking regions were designed using Primer 5.0 (Premier Biosoft, Palo Alto, CA, USA).

### Identification of Compound Mutations in *MACF1*

**Figure 1:**
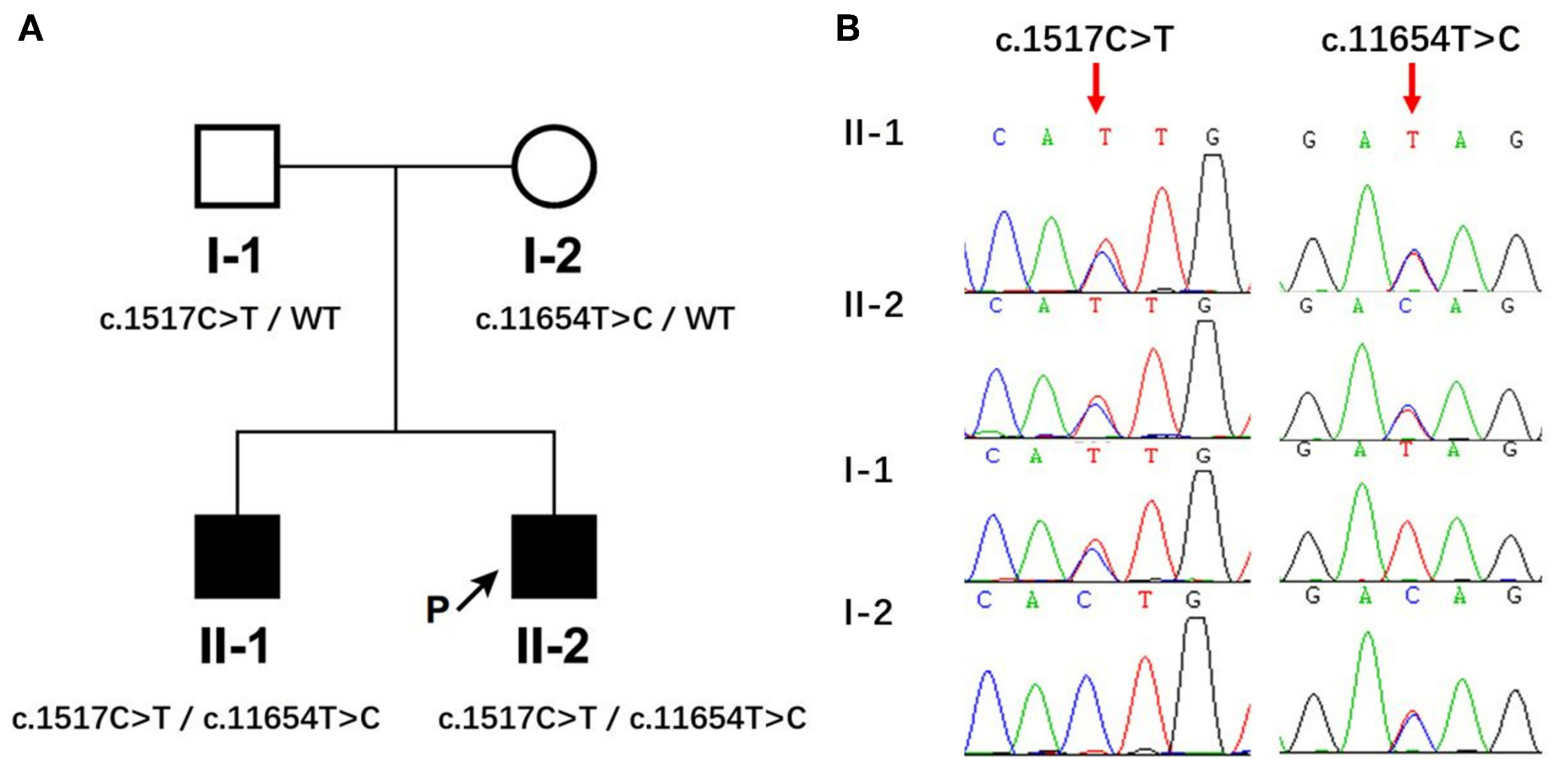
Identification of compound heterozygous mutations in *MACF1* from a Chinese family. **(A)** The pedigree with genotypes c.1517C>T and c.11654T>C at *MACF1*. Circles refer to women, squares indicate men, filled symbols represent affected patients, and open symbols represent unaffected individuals. **(B)** Partial sequences of the *MACF1* gene in the affected family. The two siblings harbored c.1517C>T inherited from their father and c.11654T>C inherited from their mother.

Whole exome sequencing was conducted in this pedigree. After filtering candidate genes using population frequency and Mendelian inheritance, two parental-derived *MACF1* mutations that co-segregated with the disease status in affected members were identified. The proband and his elder brother carried compound heterozygous *MACF1* alterations c.1517C>T (p.Thr506Ile, paternal) and c.11654T>C (p.Ile3885Thr, maternal) (Figure 1A). Sanger sequencing of the pedigree was performed to confirm the presence and origin of the meaningful variations in MACF1 detected by quartet WES (Figure 1B). Neither c.1517C>T nor c.11654T>C was reported in the Human Gene Mutation Database (HGMD). Thr506 was located at conserved position in human, chicken (ENSGALG00000003693), fugu (ENSTRUG00000005469), and zebrafish (ENSDARG00000028533), whereas Ile3885 was located at conserved position in human, mouse (ENSMUSG00000028649), chicken (ENSGALG00000003693), fugu (ENSTRUG00000005469), zebrafish (ENSDARG00000028533), and xenopus (ENSXETG00000007367), with Genomic Evolutionary Rate Profiling (GERP++) scores reaching 5.9 (the larger the score, the more conserved the site). The p.Thr506Ile mutation resides between the LRR4 (repeated sequence motifs) and SH3 (Src homology 3) domains of MACF1, whereas the p.Ile3885Thr mutation resides in a spectrin domain. The pathogenicity of the two missense variants was evaluated by Variant Effect Predictor (https://asia.ensembl.org/Tools/VEP). The polyphen-2 and SIFT software predicted the effect of c.1517C>T on the protein structure as benign or tolerated. However, according to Genome Aggregation Database (gnomAD), c.1517C>T was a population-specific variation only observed in East Asia with a minor allele frequency (MAF) of 0.0005 (9/17230). It fulfilled the parameters of similar clinical presentation with reported MACF1-related disorder, positive family history, segregation of the variant with affectedness in the family, and low frequency in equivalent populations. Thus, mutation c.1517C>T could be classified as “uncertain significance” with evidence of PM2 (moderate evidence of pathogenicity) and PP1 (Supporting evidence of pathogenicity), according to The American College of Medical Genetics and Genomics (ACMG). Polyphen-2 and SIFT predicted that mutation c.11654T>C (p.Ile3885Thr) would influence the protein structure of MACF1. The mutation fulfilled the criteria of PM2, PP1, and PP3 (Supporting evidence of pathogenicity); therefore, it could be classified as a variant of uncertain clinical significance. Globally, c.11654T>C is a rare variation, with a MAF of 0.000016 (4/245968). Ile3885 resides in the spectrin domain, which acts as a spacer region by separating the different functional domains at the N- and the C-termini, and variation from Ile to Thr at position 3885 might induce a change that could alter the structure of MACF1.

## Discussion

To the best of our knowledge, this is the second study on MACF1-associated neuromuscular disease and the first report of MACF1-related human genetic diseases in China. The first report by Jørgensen et al. described a proband with periodic hypotonia, lax muscles, joint contractures, and diminished motor skills, which resulted from a heterozygous duplication that covered a large part of the *MACF1* gene. The author provided a unique insight and showed that the heterozygous duplication resulted in overall decreased *MACF1* gene expression and *MACF1* was implicated in the novel myopathy, which they named as spectraplakinopathy type 1. All the affected children and their mothers were heterozygous for the duplication and suffered clinical signs similar to the proband to varying degrees, whereas unaffected siblings, their fathers, and maternal relatives did not carry the mutation (10). While the *MACF1* duplication co-segregated with the phenotype in this pedigree, the incomplete penetrance with highly variable clinical presentation rendered the inheritance pattern elusive.

We identified parental-derived, compound heterozygous missense mutations (Thr506Ile and Ile3885Thr) in MACF1 in two siblings using the whole exome sequencing. Highly similar phenotypes, including developmental delay, spastic tetraplegia, hypotonia, and joint contracture, were presented in both affected siblings. The co-segregation of *MACF1* variations and disease status in this family, the role MACF1 plays in mammalian nervous system development (14), and the overlaps of the clinical manifestations of our two patients with the previously reported neuromuscular disorder spectraplakinopathy type 1 caused by *MACF1* duplication (10) support the view that *MACF1* mutations could be causative in our pedigree. In the present study, no deletion, insertion, or rearrangement was detected after analyzing the genomic data using Integrative Genomics Viewer (IGV); therefore, we did not perform any further tests to find structural variants. This is a limitation of our study; further tests should be conducted in a future study.

In the previously reported pedigree (10), carriers of the heterozygous *MACF1* duplication shared the core symptoms but to a highly variable extent. The *MACF1* duplication was found to interfere with MACF1 mRNA and protein expression via an intriguing molecular mechanism. Whether the allele was a neomorph allele with a dominant negative function or a null allele of a haploinsufficient gene remained unclear. Our pedigree showed an autosomal recessive pattern of inheritance with clinically unaffected parents as carriers and highly concordant clinical presentation of both affected siblings. Mendelian co-segregation of the mutations suggested that *MACF1* caused this novel neuromuscular disorder following an autosomal recessive inheritance pattern instead of haploinsufficiency.

Pan-body knockout of *Macf1* in mice is embryonic lethal. In tissue-specific knockout models, MACF1 was reported to be associated with embryo development, neurodevelopment, skin integrity maintenance, cardiomyocyte response to hemodynamic overload, colonic paracellular permeability, and bone formation (5). Nevertheless, considering the clinical symptoms identified in our patients, the manifestations of cardiac involvement, epidermal disturbances, intestinal disorders, and bone diseases have not yet appeared. Whether our patients will develop these disorders will require a comparatively long period of following-up. In addition, muscle biopsy is arguably needed for the diagnosis of spectraplakinopathy type 1 (10). However, the two siblings in our study were not analyzed for morphological changes in their muscles because they were too weak to endure a muscle biopsy.

According to the HGMD, a total of seven different *MACF1* mutations have been reported up to January 2018 (Table 1). It included five missense mutations, one splicing mutation, and one gross insertion. It was suggested that mutation c.6868A>G increased the risk of type 2 diabetes and decreased serum HDL-cholesterol levels (15), whereas variations including c.7984G>A and c.11614A>G were involved in fetal akinesia (16). Moreover, many genes are comorbid for autism spectrum disorder, and mutation c.9040C>G in *MACF1* was identified to be associated with functional processes and pathways associated with autism (17). In a study of schizophrenia among a Chinese population, strong evidence suggested *MACF1* as a candidate gene for schizophrenia. *MACF1* was reported to harbor a damaging *de novo* variant at c.6289C>T in schizophrenia patients (18). In addition, duplication on chromosome 1p34.3 (chr1: 39497393–39800239, HG19), which gave rise to decreased protein expression of MACF1, was identified as a contributor to a novel myopathy named spectraplakinopathy type 1 (10). Splicing mutation c.15576+1G>A (IVS89+1G>A) in *MACF1* has been implicated in the pathogenesis of non-syndromic craniosynostosis (19). In the present study, two novel missense mutations c.1517C>T and c.11654T>C were identified. It is presumed that the nucleotide change led to the substitution of Thr by Ile at position 506 and generated an amino acid change from Ile to Thr at codon 3885. Further functional tests should be conducted to assess their pathogenicity.

**Table 1:** Summary of the variations in *MACF1* (OMIM 608271) based on HGMD.

**No**.	**Variant type**	**Mutation**	**Amino acid change**	**Heterozygote/Homozygote**	**Disease**	**Year**	**References**
1	Missense	6868A>G	M2290V	Not recorded	Diabetes type 2	2013	(15)
2	Missense	7984G>A	E2662K	Heterozygote	Fetal akinesia	2014	(16)
3	Missense	11614A>G	I3872V	Heterozygote	Fetal akinesia	2014	(16)
4	Missense	9040C>G	L3014V	Not recorded	Autism spectrum disorder	2014	(17)
5	Duplication	Incl. most of gene	–	Heterozygote	Myopathy	2014	(10)
6	Missense	6289C>T	R2097W	Not recorded	Schizophrenia	2015	(18)
7	Splicing	15576+1G>A	–	Not recorded	Craniosynostosis	2017	(19)
8	Missense	c.1517C>T	T506I	Heterozygote	Myopathy	2018	This study
9	Missense	c.11654T>C	I3885T	Heterozygote	Myopathy	2018	This study

In conclusion, our study highlighted the association of the human genetic disorder spectraplakinopathy with the MACF1 gene, contributed to the clinical spectrum of the disease, expanded the panel of *MACF1* mutations in patients with spectraplakinopathy type 1, and most importantly, indicated that the inheritance pattern of spectraplakinopathy could be autosomal recessive.

## Ethics Statement

This study was approved by Hospital Institutional Ethics Committee in accordance with the Declaration of Helsinki. Written informed consent was obtained from the parents of the patients for the collection of samples and publication of medical data.

## Author Contributions

YY conceived the study. LK drafted the manuscript. YL, JS, YaZ, YJ, and ML participated in the clinical management and data collection of the patients. YiZ performed the DNA analysis. All authors read and approved the final version of the manuscript.

### Conflict of Interest

The authors declare that the research was conducted in the absence of any commercial or financial relationships that could be construed as a potential conflict of interest.
